# “It’s about creating a sense of we”: exploring the role of civil society in intercultural communication during COVID-19 in Stockholm, Sweden

**DOI:** 10.3389/fpubh.2026.1809191

**Published:** 2026-07-20

**Authors:** Sofie Bäärnhielm, Mattias Strand, Baidar Al-Ammari, Soorej Puthoopparambil, Önver Cetrez

**Affiliations:** 1 Department of Clinical Neuroscience, Karolinska Institutet, Centre for Psychiatry Research & Stockholm Health Care Services, Stockholm, Sweden; 2 Transcultural Centre, Stockholm, Sweden; 3 Global health and Migration Unit Department of Women’s and Children’s Health, Uppsala University, Uppsala, Sweden; 4 Faculty of Theology, Uppsala University, Uppsala, Sweden

**Keywords:** civil society organisations, COVID-19, health crisis management, intercultural communication, language, vaccination

## Abstract

**Introduction:**

Civil society organisations (CSOs) can play an important role in crisis management and facilitate effective communication and crisis response. To improve capacity for intercultural health communication in future health crises, it is crucial to learn from CSOs’ experiences during the COVID-19 (SARS-CoV-2) pandemic and from their cooperation with healthcare systems. This study aims to gain insights into CSOs’ experiences of intercultural health communication during the pandemic in two disadvantaged areas in Stockholm, Sweden. We also wanted to explore healthcare staff’s experiences of collaboration with CSOs.

**Methods:**

We conducted a qualitative thematic analysis of interviews with informants from 15 CSOs with substantial local pandemic activity. Additionally, we conducted a secondary analysis of qualitative interview data from 21 informants involved in delivering intercultural health crisis communication during the pandemic.

**Results:**

CSOs emphasised the importance of effective and trusted communication, which requires more than translation. They highlighted the need for a deep understanding of how language, cultural frameworks and lived experiences shape the reception of public health messages. From the perspective of healthcare staff, the collaboration with CSOs was both necessary and transformative. The partnership with CSOs was a process of “joining forces, “in which mutual learning and shared responsibility strengthened the overall intercultural communication strategy.

**Conclusion:**

In times of crisis, CSOs have the capacity to act as community-based knowledge brokers by strengthening trusted, understandable intercultural communication and fostering a sense of community. However, CSOs are not just an extension of the public health authorities, but independent actors with a unique ability to communicate and build trust. Collaborative structures between CSOs and healthcare need to be firmly established well ahead of future health crises. Our results emphasise the importance of culturally and linguistically adapted communication as an integral part of inclusive crisis communication. These findings position CSOs as community-engagement actors in inclusive emergency preparedness.

## Introduction

Civil society organisations (CSOs) can play an important role in crisis management. Public health authorities’ collaboration with CSOs can facilitate timely and efficient crisis response (1). The World Health Organisation (WHO) defines CSOs as non-state actors encompassing a wide range of organisations, including medical nongovernmental organisations (NGOs), youth activist CSOs, and community relay organisations (1). During the COVID-19 (SARS-CoV-2) pandemic, a key resource for mitigating the health consequences was reliable communication about the virus and vaccination, understood and trusted by all groups in society (2). For effective health risk communication for vulnerable populations in a pandemic, a body of literature addresses the importance of culture, language and community engagement, participatory approaches engaging communities and the central importance of building trust in authorities’ communication (3, 4). In many parts of the world, risk communication campaigns during the COVID-19 pandemic targeting linguistically and culturally diverse groups failed, especially at the outset (5). White et al. (5) point out that the campaigns seem to have overlooked the social context, ignoring the fact that perceptions of health are deeply cultural. They emphasise the importance of providing information in a language and cultural framework that people can understand and relate to. In health crises, WHO (6) emphasises the importance of identifying people whom the community trusts and building relationships, including them in decision-making to ensure interventions are collaborative, contextually appropriate, and community-owned. The theoretical framework for health crisis communication outlined by the United States Centres for Disease Control and Prevention (CDC), emphasises the phased, situation-specific, and culturally sensitive nature of effective outreach and community engagement with vulnerable populations (3, 4). Building on this conceptual model, Seeger et al. (7) underscore the importance of adapting messages to take into account the information needs, cultural preferences and existing knowledge of specific audiences, including their perception of their ability to carry out recommended actions (self-efficacy) (7). Crisis psychology, acknowledges that people tend to process and act on information differently under stress than they would in non-crisis contexts (4), and this can be strengthened by iterative communication strategies that actively engage vulnerable populations and acknowledge their cultural orientations, priorities, and lived realities (3).

Several crisis communication studies during the COVID-19 pandemic have shown the importance of trust and community engagement, and the close link between them (8). Community engagement has become an increasingly influential concept in public health policy and practice, and two dimensions of community engagement are particularly salient. The first concerns the stage of the research or intervention process at which communities are allowed to become involved (9). The second dimension concerns the degree of involvement, conceptualised along a continuum from minimal to maximal engagement: from mere information provision, through consultation and genuine partnership, to delegated power and, at the far end, citizen control. A thoroughgoing theoretical elaboration of this latter pole is Mohan Dutta’s so-called culture-centred approach (10), which positions communities not as targets or even partners in health communication, but as the primary agents in defining health problems and generating solutions, though the approach remains more influential as a critical framework than as an applied model in mainstream public health practice. This orientation also aligns with asset-based approaches to community development, foregrounding instead the capacities and resources already present within communities (11). Low-income communities may have community capacity, but this is often unrecognised, underdeveloped, or underused (3). During the COVID-19 pandemic, CSOs were found to be able to contribute to delivering clear, accurate messaging to communities about COVID-19 and vaccination, and to provide social support (12).

Globally, CSOs work under very different social conditions. Kövér et al. (13) discusses government-CSO relations during the pandemic across countries with diverse socio-political and economic systems. While government-NGO relations did not deviate much during the pandemic, some countries were able to utilise the potential of the civil society, which effectively contributed to reducing the consequences of the crisis while strengthening a sense of solidarity and belonging in their communities (13). A study exploring CSOs’ response to COVID-19 in five of the most heavily impacted low- and middle-income countries (LMICs) in the Global South demonstrates the critical role of CSOs in supplementing government emergency aid responses by supporting highly vulnerable populations (12). In the Global North, high-income settings, a Canadian study Purewal et al. (14) exploring multilingual information during the COVID-19 pandemic found that community-based organisations contributed to health communication efforts and succeeded in reaching populations overlooked by mainstream communication channels. The study described CSOs as community-based knowledge brokers for people whose first language is not English.

Early in the pandemic, Stockholm—the capital of Sweden—and the surrounding areas were severely affected by the COVID-19 virus, particularly multicultural and economically disadvantaged neighbourhoods with large migrant communities. A disproportionate risk of COVID-19-related morbidity and mortality was seen among migrants in Sweden, with a particularly high risk among migrants from the Middle East and North Africa (15). This pattern, observed during the first wave of the pandemic, persisted among some migrant groups during the second wave (16). Moreover, COVID-19 mortality was probably highly underestimated among those with a migrant background (16). Rostila et al. (17) found a much higher risk of COVID-19-related mortality in most migrant groups in Sweden throughout the pandemic, especially during the early phase. A governmental evaluation of the management of COVID-19, the so-called “Corona Commission” (18), found that foreign-born individuals in Sweden were particularly hard hit by COVID-19, especially during the first pandemic wave. Further, it recognised that foreign-born individuals, especially those born in LMICs, were at increased risk of severe COVID-19. These disparities were not due to differences in socioeconomic factors or medical risk factors between foreign- and domestic-born individuals (18). The commission highlighted that different groups had had varying opportunities to protect themselves. An external evaluation of Region Stockholm’s management of COVID-19 found that neighbourhoods where many people do not speak Swedish were particularly vulnerable at the beginning of the pandemic. It concluded that the region’s communication in languages other than Swedish began too late (19). Region Stockholm is the regional administrative authority responsible for healthcare for 2.45 million inhabitants—approximately 25% of the Swedish population—and comprises 26 municipalities. To address the urgent need for effective intercultural COVID-19 communication during the pandemic, Region Stockholm undertook several measures (19, 20) and worked with many different actors (19), including CSOs (21).

Unlike many other countries, Sweden did not impose strict lockdown or curfew measures during the COVID-19 pandemic, instead relying heavily on voluntary social distancing. Sweden built this strategy on trust in two directions: citizens were expected to trust the public sector, in the form of the state, municipalities, and regions, which in turn trusted the citizens’ willingness and capacity to adhere to the recommendations (21). This approach places great demands on communication and collaboration as key tools when a trust-based society, such as Sweden, faces a deep and prolonged crisis (21). Sweden ranks among the countries with the highest levels of interpersonal trust and trust in authorities (22, 23). However, this pattern does not necessarily apply to all groups in society. For example, migrants with experience of war and state persecution are more likely to distrust the authorities (24). During the pandemic, information was provided by the government and authorities, as well as by regions and municipalities. Language became a particular challenge, and there was a lack of clarity about responsibility for communication in languages other than Swedish (21). However, gradually, authorities began translating information into languages other than Swedish.

During the COVID-19 pandemic, Swedish regions and municipalities established cooperation with CSOs that was essential to supporting authorities in reaching vulnerable groups (21). Sweden has a long history of collaboration between CSOs and authorities. In recent decades, cooperation has become increasingly formalised and structured, but has also taken various forms across different parts of the country (21). At the beginning of the COVID-19 pandemic, in spring 2020, the National Organ for Dialogue and Consultation between Government and Civil Society (NOD) was assigned by the Swedish government to assist in coordinating civil society efforts during the pandemic (25). Learning from communication efforts targeting multilingual populations in disadvantaged areas during the COVID-19 pandemic will help us better prepare for future health crises and identify areas for improvement. However, there is a knowledge gap regarding the experiences of CSOs that worked in multicultural communities and engaged in intercultural communication during the COVID-19 pandemic, particularly regarding collaboration between CSOs and healthcare systems in Sweden and other Global North contexts. To inform future health crises, it is important to draw lessons from CSOs’ experience with intercultural COVID-19 communication during the pandemic and their cooperation with healthcare systems.

The overall aim of this study is to gain insights into CSOs’ experiences with intercultural health communication during the COVID-19 pandemic in two disadvantaged multicultural areas in Region Stockholm. An additional aim is to take part in the experience of collaborating with CSOs from the perspective of healthcare staff involved in intercultural healthcare communication during the pandemic. The purpose is to strengthen intercultural health communication capacity for future health crises.

## Materials and methods

### Study design and choice of method

This study is part of a broader empirical research project that appraises intercultural communication strategies employed in Region Stockholm during the COVID-19 pandemic, focusing on the multicultural and socioeconomically underprivileged areas of Järva and Södertälje (20, 26). Data from the various groups interviewed are presented in separate articles. For this study, we used a qualitative research design comprising two datasets: one based on interviews with CSOs in the two communities, and the other based on interviews with staff involved in designing, organising, and delivering public health crisis communication during the COVID-19 pandemic (hereafter called staff).

### Study settings

The study sites are two multicultural, socioeconomically disadvantaged municipalities in the Stockholm metropolitan area: Järva and Södertälje. Both places have a high proportion of migrant residents and were severely affected during the COVID-19 pandemic, particularly in the early stages. Both areas are administratively part of Region Stockholm and include several local healthcare facilities. Södertälje has its own local hospital, while Järva’s nearest hospital is in central Stockholm. The two municipalities also have differences. Järva has a relatively young population with approximately 90,000 inhabitants. The population reflects various waves of migrants and refugees who have arrived in Sweden over the past half-century. The area is ethnically very heterogeneous, with large Arabic- and Somali-speaking populations. Södertälje is a separate municipality south of Stockholm, with approximately 100,000 inhabitants. The municipality has a long history of immigration, currently characterised by the presence of migrants from the Middle East. Södertälje is the centre of Assyrian/Syrian presence in Sweden, and the Assyrian/Syrian population accounts for at least one-third of the total population.

### Sample and sampling

For both datasets, purposive sampling (27) was used to obtain a variety of experiences. For the CSO dataset, 15 key persons, eight women and seven men, from CSOs who had been locally active during the COVID-19 pandemic were included. We used the construct of information power (28) to determine the sample size. We identified the informants and CSOs using our local knowledge, with support from locally trusted key persons. We balanced the sample with respect to the representation of both areas, faiths, and primary language profiles. We clustered the interviewed organisations into three groups: only locally community active CSOs (*n* = 6); CSOs working locally and more broadly, using language, advocacy, and web-based media platforms (*n* = 5); and religious communities (*n* = 4). The informants and organisations are pseudonymised and are hereafter referred to as L-CSO (locally based), LW-CSO (language, advocacy, and/or web-based), and R-CSO (religion-based) congregations. All approached CSOs agreed to participate. Data from CSOs were collected specifically for this study. However, for the staff component, we used a dataset from a previous study that explored staff outreach to minority communities and how public health crisis communication was designed, organised and delivered in Stockholm during the COVID-19 pandemic (29). The staff dataset comprised in total 21 individual interviews. For the present study, all 21 staff interviews from this companion study were screened, and every passage in which staff discussed CSOs was extracted to form a CSO-focused sub-dataset, with the CSO interviews serving as the primary dataset. The CSO informants held central or representative roles in their organisations, including leaders of the faith-based congregations, and covered both areas and the main language profiles. The 21 staff represented different levels and functions in Region Stockholm’s pandemic communication (29).

### Data collection

We conducted interviews with the CSOs between February 2021 and August 2023. The interviews lasted between 12 and 60 min, with an average of 40 min. Most interviews were face-to-face in local communities; five were conducted digitally. Nearly all interviews were conducted in Swedish (*n* = 12); one in Arabic, translated into Swedish by the interviewer; two in Assyrian/Suryoyo, with Swedish mixed in, and fully translated into Swedish by the interviewer. We used a semi-structured interview guide based on existing literature on intercultural communication during the COVID-19 pandemic and on findings from our previous studies exploring residents’ experiences in Järva and Södertälje. We used the interview guides flexibly, following the informants’ lead and using prompts to encourage further elaboration and clarification. With staff, individual in-depth interviews were conducted with eight participants, and 13 participants were interviewed through three focus group sessions (29).

### Data analysis

For both data sets, the interviews were transcribed verbatim. For the CSO dataset, the Arabic-to-Swedish translation was verified by the interviewer together with the interviewee and a bilingual resource person. All interview transcripts in which healthcare providers discussed CSO were extracted to create a new sub-dataset. The two data sets were analysed separately, and for both, a thematic analysis was carried out following Braun and Clarke’s guidelines (30, 31). The thematic analysis framework was used to analyse the interview data, as this approach reflects the idea that knowledge and meaning are created through social interactions and experiences, which are necessarily interpreted through the lens of cultural, contextual and individual level factors (30, 31). Our analytical approach was explorative and inductive. It was not based on pre-established theoretical conceptions. We started with familiarisation with the data, generating codes, constructing themes, reviewing potential themes, defining and naming subthemes, and producing the report (30, 31). For the CSO dataset, coding was first performed by BAM and discussed with SB. Thereafter, SB reviewed the coding and analysis again, and the findings were then discussed with the whole research group. Themes were revised until consensus was reached. For the staff dataset, coding was initially carried out by SB, then discussed with MS and BAM, and subsequently with ÖC and SJP. The meaning units, themes and subthemes for both datasets were revised several times until consensus was reached to address rigour, reliability and dependability (32), as well as the use of NVivo12 software (33).

### Ethical considerations

The Swedish Ethical Review Authority granted ethical clearance (No. 2022–01637-01). This study was conducted in accordance with ethical standards in research (The Declaration of Helsinki 1975). We collected written informed consent from most informants after providing both written and oral information. For some informants, we documented consent digitally. Participation was voluntary and could be withdrawn at any time. Potential risks for the informants could include breaches of integrity due to the collection of sensitive data, and the risk of recognition, as some CSOs were key persons in their respective communities and could easily be recognised. We tried to minimise the risks through several means: when transcribing the data, the transcripts were pseudonymised and information that could identify the informants was omitted or changed; we present the results thematically on group level, rather than individually; the results presented exclude characteristics that could reveal information about the participants; we have been careful in our description to avoid stigmatisation of the targeted groups; we have kept code lists separate from coded data. The CSO interviews and the staff dataset (29) are part of the same research project and are covered by the same ethical approval. The original informed consent included secondary analysis of the interviews within the project. The study protocol has been preregistered on the Open Science Framework (osf.io/rt47j).

## Results

The results from CSO and staff interviews are presented separately, as staff experiences of collaboration with CSOs are intended to complement the CSOs’ own narratives.

### CSOs result

The CSOs’ experiences of intercultural health communication during the COVID-19 pandemic involved multitasking within a network of contacts. Early in the pandemic, CSOs took the initiative to facilitate intercultural communication with residents and authorities, drawing on contacts with and knowledge of their target groups. The first theme offers a general perspective on the nature of CSOs’ intercultural communication work. The following four themes deepen the understanding of the CSOs’ communication.

#### Theme 1: promptly initiate multidirectional intercultural communication and social support

For the CSOs, intercultural communication was a multitasking process. It included informing the local communities about COVID-19, collaborating with authorities and media, raising an alarm about the infection situation, advocacy, and counteracting misinformation. As a result, CSOs were active in a variety of networks. Two subthemes are included.

##### Subtheme: conveying information, raising alarm and initiating advocacy

When the pandemic began, CSOs found it essential to respond quickly and convey information in more languages than Swedish to the communities:

*“We were actually ahead of the Södertälje municipality and many other actors in the field in reaching out with information in several different languages.”* (LW-CSO).

The CSOs also realised that information had to be adapted to the target groups and that different communication approaches were required.

Informants reported how the first major COVID-19 outbreak in Sweden occurred in the Somali community in the Järva area. The CSOs were among the first to learn about this, as they were contacted by healthcare workers at hospitals serving the area. The CSO representatives felt an enormous responsibility in handling this information, as they feared that conveying it could create panic or lead to the area being completely sealed off. Through their networks, the CSO raised the alarm about widespread infection rates and mortality, and promoted media coverage of the situation to mitigate the rapid spread of the virus:

*“We got together and invited the authorities, enabling them to inform the civil society, mosques, churches, and local community*.” (L-CSO).

The CSOs collaborated with each other, facilitated by long-standing pre-pandemic relationships between local CSOs and religious congregations. They initiated advocacy, paying attention to the consequences of social situations such as overcrowding in housing and on public transport. The CSOs divided the work between them.

*“Some organisations engaged in extensive monitoring of the pandemic’s impact, including economic and social consequences.”* (LW-CSO).

##### Subtheme: different approaches to counteracting misinformation

All interviewed CSOs were actively involved in countering COVID-19 misinformation but did so in different ways depending on the nature of their organisation. For some, this took place mainly through discussions with individual community members, for others, through media platforms. One organisation, working with a language-based social media platform, focused on counteracting false rumours, including those that downplayed or exaggerated the danger of COVID-19, or conspiracy theories. In doing so, they also found it essential to allow space for criticism and doubt:

*“We should give space even to people who are doubtful—they may have a point, and we should listen, as there is some logic in what they say, we listen. We have not boycotted anybody; we let everyone be heard.” (LW-CSO).*

Concerning vaccination, there were different views among the CSOs. Most argued actively in favour of vaccination, but there were also doubts (see below).

#### Theme 2: taking on the challenge of translating and adapting pandemic information

This theme explores how CSOs rose to the challenge of translating and adapting language and information, and of demonstrating creativity in communication strategies. The theme includes two subthemes.

##### Subtheme: working towards a comprehensible language across multiple languages

Providing information in multiple languages was considered crucial to ensure that COVID-19 communication was understandable and that critical information reached all concerned.

*“…. It is not a right to have everything translated into one’s mother tongue, but critical civic information must reach all citizens of a country without exception*.” (LW-CSO).

Many CSOs translated and adapted the authorities’ COVID-19 information into different languages. The translation was deemed necessary, particularly to reach older people and new arrivals with limited command of Swedish. However, comprehensible communication did not only include linguistic translation but also many other aspects of language use. A significant challenge was translating and conveying the meanings of scientific and complex concepts such as “pandemic” and “restrictions” into languages where these terms were unfamiliar. To overcome translation difficulties, CSOs used everyday examples instead of technical terms. Even for Swedish speakers, the authorities’ messages were found to be challenging to understand due to their overly academic, technical level. An example is the officially used term “restrictions,” which can imply varying degrees of obligation:

One informant was asked to give examples of words that were difficult to translate:

*“For example, the word pandemic. Then the name of the disease corona…. Then there was the question of when to discuss restrictions and what they mean. Was it forbidden? Some interpreted it that way. ‘Can we not go out now?’ There are words like that. And then some difficult words when you talked about the body parts*.” (LW-CSO).

##### Subtheme: creativity in adapting methods and approaches to communication

For the CSOs, web-based channels such as social media and video broadcasting were essential tools but also meant new challenges and finding new approaches. Many older people lacked access to smartphones or the ability to use them and other digital platforms. The CSOs, therefore, also used what they called “traditional methods,” such as phone calls, letters, and oral information provided in face-to-face meetings. The importance of spoken language in the mother tongue was emphasised. Several CSOs invited locally well-known doctors, nurses, and experts who spoke other languages to provide information. Religious congregations became essential for disseminating information. Web-based information meant special challenges as information in other languages was not always understood by established algorithms and was sometimes misinterpreted and tagged as disinformation.

In various ways, CSOs sought to adapt COVID-19 communications to be culturally and contextually meaningful and aligned with how target groups live and think. This meant that, in addition to scientific knowledge, their communication included practical support and linked arguments to cultural or religious thought. For some CSOs, it was essential to reason in relation to religious perceptions, for others to focus on practical ways to protect and support one another:

*“We discussed with them how we need to protect ourselves and take care of each other during this time, rather than discussing why and how it came about.”* (LW-CSO).

#### Theme 3: communication based on existing relations of concern and caring

The CSOs’ COVID-19 communication was not just adapted; it was also anchored in their established relations of concern for the well-being of the target groups. The theme includes two subthemes.

##### Subtheme: pre-pandemic foundation of concern and caring

The CSOs’ relations, characterised by concern and caring, were established before the pandemic but varied across organisations. Religious communities and most locally active CSOs had a history of personal and practical concern for the well-being of their communities, web-based channels for addressing social and cultural issues and the rights of linguistic, ethnic, or religious groups, and advocacy organisations for improving social conditions and inclusion. Pre-pandemic relationships influenced communication and were considered essential to trust and acceptance of CSOs’ COVID-19 communication.

For several CSOs, it was not only individual practical and social support that was necessary during the pandemic, but also emotional support. This included a variety of approaches. For one, a telephone chain facilitated coping with loneliness during the pandemic. A web-based organisation described how they became a hub for others speaking the same language and who wanted to give humanitarian and knowledge support:

*“A kind of beautiful solidarity emerged in the XX-speaking community, which also encouraged us to continue our work.”* (LW-CSO).

##### Subtheme: communication anchored in trust and trust building

The importance of trust and continuous trust building in disseminating information was emphasised and addressed in several ways. Religious congregations found that many community members trusted what they were saying, but that it could be challenging to explain that this was also officially sanctioned information:

*“People trust information from mosques or churches. But if we said that it came from TV or radio, many people did not believe it.”* (R-CSO).

A common approach to anchor trust was through individual contacts, allowing time for dialogue and concern:

*“Sitting side by side with eye contact. [….] They gained more confidence. Really*.” (R-CSO).

For trust-building, CSOs emphasised the importance of being personal, inclusive, and respectful of people’s emotions. Web-based and language-specific CSOs had to bear in mind that many community members came from countries where media information is unreliable and under authoritarian control:

“*But the issue of gaining trust has come through time.*” (LW-CSO).

Additionally, the importance of respecting people’s knowledge, values and traditions was emphasised, as was the need to be confident enough to present facts that were not always appreciated.

#### Theme 4. contradictions, reactions, and creating a sense of community

Despite being anchored in relations of trust, CSOs also faced resistance, particularly regarding vaccination. The resistance was seen largely as being caused by fear, and creating a sense of community was considered necessary to counteract fear and resistance. Resistance was also associated with alternative interpretations of the pandemic. The theme includes three subthemes.

##### Subtheme: acknowledging emotions of fear and creating a sense of community

The CSOs observed that people’s fear-based emotional reactions contributed to resistance and conflict regarding COVID-19 and vaccination communication. Lack of clear information could cause fear and turn into anger:

“*Many people were worried about what was going to happen. They had been given the wrong information*” (R-CSO).

Congregations faced criticism when churches and mosques were closed.

*“There were a lot of protests. They are believers. They said, ‘How can they close the churches, now that it is challenging times, and we have to go and pray to God to help us?’“*(R-CSO).

To overcome fear and resistance, the CSOs highlighted the importance of acknowledging people’s feelings and of creating a sense of community through personal presence and emotional support.

##### Subtheme: alternative interpretations of the pandemic

CSOs encountered various ways to interpret the pandemic. These could include religious interpretations, e.g., that the pandemic was God’s punishment. Other interpretations of the pandemic led to ideas that vaccination was carried out not to protect but to cause harm, particularly among migrants.

*“…. some thought we just wanted to vaccinate everyone so we could turn them into zombies.”* (R-CSO).

##### Subtheme: vaccine communication as a particular challenge

Vaccine communication was a particular challenge, and vaccine resistance was often related to fear, lack of information, misinformation, various alternative interpretations of the pandemic and the Swedish approach to pandemic containment. Most CSOs were highly active in disseminating information about vaccination, whereas some were not. To overcome vaccine hesitancy, CSO informants pointed to the value of people receiving the correct information. The CSOs encountered many different reasons for vaccine resistance. One of them was a lack of understanding and trust in the authorities’ intentions behind vaccination. CSOs encountered the perception that in Sweden, the purpose of vaccination was not the well-being of the population, but rather economic purposes.

*“…they (authorities) want everyone to be vaccinated so that they can return to work more quickly and reach this level, so why should we be sacrificed? This is a test. All these things were interpreted as Sweden having chosen business as super, super important. That’s why no shops, no shopping centres can be closed.”* (R-CSO).

To overcome resistance, misunderstandings, and fear of vaccination, they emphasised the importance of facts and personal contacts.

*“It is important that you take it in several rounds, that you realise that this is serious. It is not like a normal lecture, whether I understand or not. As we repeated [the information] with nurses and doctors, I learned many things that I can now convey to others. So, the contact really helped a lot.”* (R-CSO).

There were CSOs who felt unsure about vaccination and did not feel that they could take a clear position on what people should do:

*“… to talk about the problem, about viruses and all this, we could and did, but to decide about vaccines or not, that matter we could not take a position on…”* (R-CSO).

#### Theme 5. valuing structured and personalised cooperation with authorities and media

The CSOs had contacts across many networks, from individuals in local communities to national and international contacts. Region Stockholm and other health authorities were key partners, and personalised cooperation valued. This theme includes two subthemes.

##### Subtheme: the worth of cooperation with authorities

To varying degrees, CSOs had contact with local authorities before the pandemic. Some described pre-contacts and others a lack of established contacts.

“… *they [the authorities at the municipality] come when there are elections.*” (R-CSO).

Others talked about well-functioning, established local networks, mainly social community authorities activated in a crisis. However, the pre-contacts with the healthcare system were mostly considered limited:

*“But in healthcare, they have not established any link to civil society, administrations, authorities, local authorities. That was what was missing, collaboration.”* (R-CSO).

During the pandemic, contact with healthcare organisations increased for most CSOs but not for all.

*“Civil society should be more involved. For example, our parish has 6–7,000 members, and they can be reached effectively if our church is involved. But during the pandemic, cooperation was weak.”* (R-CSO).

Often, CSOs found it challenging to understand the authorities’ COVID-19 information, especially as the restrictions were constantly changing. The CSOs pointed to the value of establishing one clear personalised information channel:

*“I had a lot of responsibility to gather information. We had to go through many different channels. I wish there had been a single source of information. We had to visit different sites and interpret the information. We had to go to* var*ious authorities’ websites to gather information.”* (LW-CSO).

Several CSOs provided examples of how contact with healthcare improved during the pandemic. In Järva, the value of regular contacts with Region Stockholm’s health communicators was highlighted.

“*Every week, she [health communicator] summarised the situation and provided information about what was happening, what we needed to consider and what we needed to do. It was great*.” (LW-CSO).

In Södertälje, several CSOs appreciated being contacted directly by the local hospital:

*“What was good was that Södertälje Hospital contacted us, as we had a good working relationship and daily contact. People came, officials who had regular contact with the hospital, to provide information in the church.”* (R-CSO).

Some CSOs had no direct contact with the healthcare institutions during the pandemic, but with individual health professionals.

##### Subtheme: criticising and appreciating authorities’ intercultural COVID-19 communication

The authorities’ intercultural COVID-19 communication was both criticised and appreciated. Initially, the authorities were, as described, found to be unprepared and far too slow in providing information in languages other than Swedish. Additionally, the way of communicating was perceived as hard to understand and poorly adapted to the population.

*“Yes, there were such major shortcomings in how society handled this. And it became so clear how big the differences are between the poor and the rich in this society.”* (LW-CSO).

The initial lack of understandable communication was seen as contributing to increased COVID-19-related morbidity and mortality:

*“Another week passed, and during those two weeks, several people died, and we buried them.”* (R-CSO).

However, the lack of translation was considered to have been improved over time, but still difficult for many in the local areas to understand and targeted mainly other social groups in society.

*“Once more, the information was for the elite. It was not for the ordinary public—those who cannot read between the lines and do not understand the language, the terms ‘restrictions’ and ‘recommendations’.”* (R-CSO).

For effective intercultural communication during health crises, several CSOs emphasised the importance of feeling included in the wider Swedish society and of creating a shared community sense of ‘we’ in crisis management. One informant formulated a perceived experience of not being included:

*“Here [referring to the local area] it’s more about emotions. People want to feel seen and heard, and I think many have missed that. [….] It’s about creating a sense of we, we are doing this together. I think that is something that did not work.”* (LW-CSO).

Later in the pandemic, several CSOs appreciated that Region Stockholm tried to share information in languages other than Swedish and that this was done through personalised contact with residents rather than only through written texts.

CSOs described how they were asked to help authorities in getting public health messages across:

*“We were sometimes a sounding board for messages, since we have a keen sense of what works and what does not work in the target group.”* (LW-CSO).

When authorities started using health informants who speak different languages working in local communities, this was appreciated, enhancing understanding, creating a sense of inclusion and motivating community members to get vaccinated:

*“When they meet people who speak their own language, it works well. Many were persuaded to get vaccinated. Also, how to protect yourself, what you can do, and what you can contribute. I think the information they provided about this was good.”* (L-CSO).

##### Subtheme: media contacts bring both benefits and risks

Mass media, such as the press, radio, and television, were considered essential in drawing attention to the situation in the local communities and to CSOs’ work during the pandemic. Contact with the press was mainly perceived as positive:

*“The media was in Södertälje a lot, both regional and national. […] I felt that the media contacts were good. It made us feel that we are part of this society, that we take our responsibility.”* (L-CSO).

However, media contacts could also be problematic, as community members had felt singled out and blamed for the spread of disease:

*“We felt that we had to defend ourselves, that we needed to explain why many people had fallen ill and died. We did not want to be linked to the mass spreading of the virus, as it is not positive news.”* (LW-CSO).

#### Theme 6. preparing for future health crises - embracing diversity

Through their close relationship with people and their trust, CSOs found that they could contribute to understanding and facilitate communication.

*“Civil society is an important factor. They are rooted in the neighbourhoods. They work voluntarily with children, families, through mosques, sports, culture […], and they are a channel to reach people. It’s important to use that channel.”* (R-CSO).

The CSOs emphasised their own special capacity to reach the population:

*“Now we see how important civil society really is. On the one hand, if you expect the authorities to fix everything when such a crisis or chaos breaks out, no authority in the world would be able to do it. No government alone could cope with it. So civil society must come in and play its part. But we also saw that when many actors came forward, things happened.”* (L-CSO).

For handling a health crisis, several CSOs emphasised the importance of embracing diversity and inclusion in leadership and crisis management:

*“What we pointed out at that time [in the pandemic] was that diversity and languages must be included in authorities’ leadership.”* LW-CSO.

Inclusion was seen as helpful for authorities to get to know community populations and thus facilitate effective communication and trust. A substantial problem and source of frustration for several CSOs was economic weakness. For CSOs active in socially underprivileged multicultural areas to be able to contribute to future health crises, informants pointed to the economy as an essential reality to consider:

*“…. civil society is in many ways treated in a very unfavourable way. How should I say: You have to invest in civil society. Because civil society is actually strong in this neighbourhood, against all odds.”* (LW-CSO).

The CSOs stressed the importance of preparing for future health crises through long-lasting, structured, collaborative contacts. CSOs found that they had an essential role during the pandemic including to create shared sense of community.

### Region staff result

Early in the pandemic, it became clear to staff in Region Stockholm working with COVID-19 communication that they needed local support to overcome communication barriers in underprivileged multicultural areas where many spoke languages other than Swedish. Concerning healthcare staff’s experiences of collaboration with CSOs, we identified three themes.

#### Theme 1: facing intercultural communication barriers and lack of trust

Staff quickly recognised the need for COVID-19 information in more languages than Swedish. They also identified other challenges and barriers, such as the need for oral information and the importance of interactive dialogue, as well as the lack of trust among many residents in information from the authorities.

*“... we come as an authority and say something and try to gain the trust of people who do not trust authorities. It’s a barrier.”*

The staff realised that they needed to find ways and collaborators to reach the communities, and this stimulated staff creativity in trying to understand the challenges and to find new ways to reach out:

*“… I want to understand why people might not want to listen to … or not be reached by the information (about COVID-19). It is important that you find the right ways in ….”*

#### Theme 2: joining forces with CSOs to overcome communication barriers

In efforts to reach out to local communities, Region Stockholm’s communication staff felt greatly assisted by other government bodies, such as municipalities, the County Board (Länsstyrelsen), and the Swedish Agency for Support for Faith Communities. These authorities provided contact information with locally active CSOs. The Region was also directly contacted by several CSOs early in the pandemic with information on local situations, needs, suggested actions, and ways to improve communication. The contact with CSOs and their localised knowledge about target groups and networks was found to be very important and valuable for reaching out to the communities. CSOs helped to enhance the credibility and trustworthiness to the authorities’ communication.

*“But without them [CSOs], without being able to join forces and do it together, the Region would not have been a credible part of things.”*

For staff, this new way of working and collaborating with CSOs facilitated adapting and improving the relevance of their communication in the fast-changing pandemic situation. Referring to the importance of dialogue with local communities, one informant said:

*“But we needed this knowledge of how it works in practice, in reality, every day.”*

The collaboration with CSOs and their localised knowledge was described in terms of help for the Region “*in keeping our ear to the ground.”*

The great importance of contact with faith communities was stressed. Staff from the Region initiated contact with several local faith communities. This was particularly important considering the need for trust in the messenger conveying public health information:

*“…. we had a lot of contacts through different faith communities. Perhaps in some neighbourhoods, they might listen more to what is said in their church or their mosque….”*

In particular, with respect to vaccination, religious leaders played a significant role. One informant described the nature of the cooperation:

*“So, if you take the vaccinations, it was absolutely crucial that the faith leaders could sometimes stand hand in hand with the Region’s infection prevention and control physician in the church in Södertälje during the festival and talk about COVID-19 and how important it is to get vaccinated. It would never have been possible otherwise, if it were not sanctioned and available. Or with the imam at Medborgarplatsen [Stockholm central mosque].”*

The informant described how Region Stockholm needed to adopt an approach of not necessarily being the visible messenger but rather supporting CSOs with facts and documentation.

#### Theme 3: good practices for future crises

The staff described the collaborative approach of CSOs during the pandemic as a novel experience. Before the pandemic, there were already some contacts with CSOs, such as the regional office for infection prevention and control (Smittskydd). However, collaboration with CSOs increased significantly during the pandemic, and the Region took on a new role.

*“You cannot build this type of structure during a crisis. It should be there already. And I think that is also a lesson we have learnt that you need to have a structure where you interact with the population and have this type of dialogue.”*

The staff described how Region Stockholm both improved and established a more structured, collaborative approach with local communities and CSOs. The great importance of continuing this for handling future crises was emphasised. For the Region to have trusted communication, an informant emphasised the importance of their staff being active locally, rather than disseminating long written translations. Despite the positive experience, staff were also self-critical and felt they should have gone even further in working with CSOs during the pandemic:

*“Yes, but I think they are vital [referring to CSOs] and that we did not fully ask for help or give the help that we could have given and received.”*

In the multi-language communities, CSOs were found to be important collaborators and cultural brokers in conveying health messages.

## Discussion

This study, exploring CSOs and healthcare staff’s experiences of intercultural COVID-19 communication during the pandemic, is part of a broader empirical research project appraising intercultural communication strategies employed in Region Stockholm. The focus was on two multicultural and disadvantaged areas. The results are based on two datasets: the primary dataset comprises interviews with CSOs, and the additional dataset is a secondary analysis of interviews with healthcare staff involved in intercultural communication. Early in the COVID-19 pandemic, CSOs took it upon themselves to contribute to multi-way intercultural communication by leveraging their contacts and knowledge of their target groups. They developed their own approaches to communicating and forging contacts with health services and other authorities. The CSOs were not merely passive recipients of public health directives but emerged as proactive agents of communication, advocacy, and trust-building. They never became a mere mouthpiece for authorities but maintained an independent profile, including providing feedback to authorities, initiating initiatives to improve intercultural communication, and advocating. Their early and multifaceted engagement, often preceding formal authorities’ responses, underscores their capacity to act as community-based knowledge brokers in times of crisis. An example of this is how the CSOs brought public attention to an early internal hospital alarm about high infection rates of the virus in Järva (19, 34). From the perspective of healthcare staff, the collaboration with CSOs was both necessary and transformative. Staff recognised early on that language barriers, lack of knowledge about the recipients, and mistrust hindered the effectiveness of their communication efforts. The partnership with CSOs was described as a process of “joining forces,” in which mutual learning and shared responsibility strengthened the overall communication strategy. Staff reflected on the need for greater responsiveness to CSO input and more proactive engagement.

A central theme emerging from both datasets is the importance of language and cultural adaptation in health crisis communication. Informants emphasised that effective communication required more than linguistic translation. It demanded a deep understanding of how language, cultural references, and lived experiences shape the reception of public health messages. Discussing the unequal effect of the pandemic, the earlier mentioned governmental “corona commission” points out that the mitigating measures taken in Sweden were often better suited to a well-educated middle class with good opportunities to protect themselves against infection, navigate the healthcare system and work at home and that “… *it is the responsibility of public authorities to design measures for the whole population”* (18). For effective pandemic health risk communication, Vaughan and Tinker et al. (3) emphasise the importance of meeting the specific communication needs of all populations, especially those most vulnerable to the risks and most likely to experience communication gaps. This includes adapting and translating information, preparing for emergencies, and interacting with community organisations. Residents in the two areas experienced discrimination and even being singled out as responsible for the spread of the COVID-19 virus (26). For crisis management, several CSOs emphasised the importance for residents in the communities to feel included in the wider Swedish society, creating a shared community sense of ‘we’, and that this had failed during the COVID-19 pandemic. This can be seen as minorities wishing to be included in a broader collective Swedish identity, but that was not how it turned out during the pandemic. The sense of exclusion was heightened, given that the minorities we studied had fled countries where they already had a long history of societal exclusion and strong mistrust of authorities (26).

Trust building emerged as a foundational element for intercultural communication to reach out, as many residents in the studied communities were reported as often having limited trust in authorities (26). CSOs were embedded in the social fabric of these communities and were therefore perceived as credible and caring messengers (26). Their communication was anchored in long-standing relationships and a demonstrated concern for community well-being. These relations of trust enabled them to bridge the gap between public health authorities and residents, particularly in contexts where institutional trust was low. The findings on the significance of cultural adaptation in communication and trust building align with those from our other COVID-19 communication studies in these areas. Exploring experiences of non-native Swedish-speaking residents regarding authorities’ COVID-19 communication, we found that understanding authorities’ COVID-19 and vaccine communication and perceiving trustworthy sources of information were difficult for residents (26). The study highlights the significance of language and community participation for promoting comprehension of authorities’ communication (26). This is also in line with the experiences of the Region’s health informers working locally in disseminating information (20) and those of health communicators staffing a national multilingual telephone line during the pandemic (35). The importance of trust was also evident in a German study which investigated the factors that explain the status of people’s vaccination against the SARS-CoV-2 virus (36). The study found that trust in specific institutions (e.g., medical experts and authorities) was positively associated with vaccination status (36). The importance of trust for communication to reach out has been verified in several COVID-19 studies (8, 37, 38). With regard to vaccination, the literature suggests that migrant populations generally have a higher burden of vaccine-preventable diseases and lower immunisation rates (39). A Swedish interview study with Somali and Syrian immigrants revealed that, rather than being opposed to COVID-19 vaccination, they wanted more information before making a decision, ideally from a trusted peer, such as a religious leader or community member, or a healthcare professional (40). Also, Svallfors et al. (41) highlight the importance of establishing trust in healthcare providers and government authorities, and of providing adequate information about vaccination, for groups facing the greatest barriers to care. This enables informed decision-making. These findings from Sweden are in line with international studies showing that the most frequent factors influencing COVID-19 vaccine hesitancy were concerns about vaccine efficacy and safety (42).

The emotional dimension of the pandemic was another critical factor in our study. CSOs encountered widespread fear, confusion, and grief, which were sometimes considered as resistance to public health measures, especially vaccination. Rather than dismissing these reactions, CSOs engaged with them empathetically, offering emotional and personalised support and fostering a sense of community. Such hesitancy should be understood as situated and reasoned rather than as mere misinformation. As we found among residents in the same communities (26), it was shaped by legitimate social, structural and political factors, including histories of exclusion and mistrust carried from countries of origin (24, 26) and the fact that mitigating measures were often better suited to a well-educated majority (18). Avoiding a binary of correct authorities versus misinformed communities, our staff data (29) also point to gaps in the authorities’ own communication, underlining that institutional trust is central to vaccine uptake (23, 24, 36). In practice, the CSOs took it upon themselves to act as community cultural brokers for intercultural COVID-19 communication. The concept of cultural brokers has a long history in anthropology, referring to go-betweens who mediate and translate between culturally distinct realties for reducing conflict or producing change in relationships (43). With somewhat shifting roles, cultural brokers have been introduced in medical settings (43). Our study shows the value of CSOs as cultural brokers in crisis management for supporting understanding of authorities’ crisis communications and trust in the measures taken. Furthermore, our results clearly demonstrate that effective intercultural COVID-19 communication could not have been achieved solely through the dissemination of translated information; it was necessary to consider the concerns, emotions, fears, and genuine challenges experienced by residents. In the face of the next health or social crisis, advanced prepared culture brokers could make a significant contribution to intercultural communication rooted in community engagement. Dutta’s (10) culture-centred approach positioning communities not as targets or partners in health communication, but as the primary agents in defining health problems and generating solutions, could be a useful approach in preparing future crisis management to be inclusive. Our findings point to CSOs as possible key community resources in delivery, intervention design through feedback loops and preparing for the next health or civil society crisis. In our study, crisis management based on planned inclusive community engagement seems to contribute to foster a sense of community and a sense of a broader shared “we” among exposed groups. This approach can also counteract the spread of rumours and stigmatising narratives blaming those at high risk of exposure.

### Implications for good clinical praxis in intercultural health crisis communication and organisational learning

In our study, CSO informants and healthcare staff described a shared creative learning process that emphasised the importance of cooperation in intercultural health crisis communication during the COVID-19 pandemic. They emphasised the importance of having collaborative structures in place before a crisis breaks out. Our findings align with the theoretical framework for health crisis communication outlined by CDC, centred on the necessity of building trust in times of crisis and the importance of culturally sensitive outreach and community engagement with vulnerable populations (3, 4). Our findings show that underserved groups must be included as an integral part of overall crisis management efforts to both foster reciprocal understanding between stakeholders and a shared sense of collective action, reinforcing that “we are doing this together.” Our study suggests that the ability to understand precautionary and safety measures in health crisis communication largely depends on the capacity to interpret information within familiar linguistic, cultural, and social frameworks. It also demonstrates the important role that CSOs can play in promoting understanding and acceptance of communication within local communities. In high-income countries, societies with higher levels of volunteering have been found to be less severely affected by the COVID-19 pandemic (44). A systematic literature review about the roles of CSOs across diverse crises and phases of crisis and disaster management reveals that CSOs primarily perform three key roles: protecting human life, facilitating societal and individual resilience and recovery, and advocating for affected communities and individuals (45). For vulnerable groups, our findings emphasise the importance of preventive strategies that build trust and inclusion, and position CSOs as integral actors in public health emergency responses. The CSOs’ ability to adapt communication, build confidence, and address emotional and contextual factors makes them uniquely suited to reach vulnerable populations. WHO underscores that partnering with established CSOs can help engage communities to respond to a public health emergency (1). However, the CSO’s role as a communicative knowledge broker cannot be taken for granted in the event of a future health crisis. Looking at religious organisations’ desired role in times of national crises and disasters and the actual role taken by local congregations in Sweden during the COVID-19 pandemic, Lundgren and Fransson (46) found a willingness to take part in the event of a disaster and crisis, but also point out that this willingness is not unconditional (46). They found that considerably fewer were willing to provide information about the vaccine compared to information about the pandemic and concluded that the Swedish government cannot expect minority religious communities to perform duties for them unfalteringly, even though there is an underlying desire to help (46).

For future preparedness, it is essential that public health systems institutionalise mechanisms for collaboration with CSOs, include them in crisis planning and leadership and ensure that these relationships are maintained and nurtured beyond the immediate crisis—this includes embracing diversity in crisis management but also to understand and accept the limits of what the CSOs can and want to contribute with. Many CSOs operated with a volunteer base and limited resources, and their early engagement should not be taken for granted, as the willingness of community and faith-based organisations to assist is real but conditional (46). Sustained collaboration must therefore safeguard CSO autonomy and avoid instrumentalising or over-relying on them, attending to the power asymmetry between authorities and community organisations (1, 12, 13). Framed in terms of community resilience, CSOs functioned as social capital and adaptive capacity. By building trust, fostering a shared sense of “we,” and counteracting stigma and rumours, the CSOs strengthened the communities’ capacity to respond to the crisis, consistent with our companion studies in the same areas (20, 26, 35).

For Region Stockholm, our findings are in line with the previously mentioned external evaluation of the Region’s handling of the COVID-19 pandemic, which concluded that to prepare for future crises, the Region needs to develop a strategy and a plan to communicate to groups where many speak languages other than Swedish (19). The extent to which the learning process about how to reach out with intercultural communication through individuals in CSOs and staff in Region Stockholm during the COVID-19 pandemic implies a shared organisational learning process within their organisations, we cannot tell from this study. The concept of Organisational learning (OL) refers to organisations’ ability to transform individual knowledge into organisational knowledge (47). OL enables organisations to adapt to dynamic environments by transforming individual insights into collective knowledge. This implies a systematic process of acquiring, retaining and sharing knowledge throughout an organisation. Although healthcare is a knowledge-based sector, there is a risk that knowledge gained from infrequent situations remains as individual tacit knowledge and thus does not fully contribute to organisational learning. Accordingly, it may not affect future crisis management. This study highlights the significance of organisational learning from intercultural communication experiences gained during the COVID-19 pandemic in healthcare.

### Trustworthiness and reflexivity

In this study, we have explored CSOs’ experiences from their own perspective. Three of the authors are familiar with the areas (SB and BAA with Järva, and ÖC with Södertälje) and thus have insider knowledge of the CSO’s local pandemic activities. Two of the authors had a more neutral position. To enhance trustworthiness and credibility, several measures were taken. The entire research team read through all the interview transcripts in depth. Systematic coding was conducted using NVivo 12 software, which facilitated data management. Double coding was performed, with BAM coding the interviews first, followed by SB. The codes were discussed, discrepancies were identified, and the coding framework was refined, thereby enhancing reliability. These discussions led to consensus on the final codes, thereby mitigating biases. The staff findings are a secondary analysis of data to explore the experiences of staff involved in intercultural COVID-19 communication (29, 35). These interviews provided a wealth of information on cooperation with CSOs.

This study has its limitations. We interviewed 15 CSOs that were locally active during the COVID-19 pandemic but may have overlooked others that were important. Although no CSOs declined to be interviewed, we may have missed those with different views on the pandemic and cooperation with health authorities. One limitation we have described is that, in order to protect the informants’ anonymity, we cannot provide more detailed information about the various CSOs, as this would risk identifying the informants. This is also the case for informants from the Region. CSO representatives are often not officially organised or appointed but are more often self-appointed. They have their own profession and skills, doing their best in representing their community. This obviously reflects their personal or collective ideology and interests, and possibly their goals or agenda. We have not examined the COVID-19 communication provided by the CSOs. However, based on the information received from residents (26), health authorities (29) and health informers (20) working locally, there is no indication that the CSOs we interviewed disseminated information that contradicted that of the authorities (29). As we have not sampled participants systematically or randomly, the output is not generalisable. Still, the data and our themes give a broad picture. Thus, a larger, more diverse, and more systematic interview sampling could enhance the external validity of our results. One strength of the study is that it comprises two datasets analysing the same situation.

## Conclusion

The experiences of CSOs and Region Stockholm healthcare staff collaborating on intercultural health communication during the pandemic demonstrate CSOs’ capacity to strengthen trusted, understandable communication, provide social support and foster a sense of community in times of a health crisis. The CSOs can act as community-based knowledge brokers. Our results show that the CSO was not merely an extension of the authorities but an independent actor with a unique capacity to communicate and build trust during the COVID-19 pandemic. The study highlights the value of structured cooperation between CSOs and healthcare and the need for community preparation to be in place before a future health crisis. Our results emphasise the importance of culturally and linguistically adapted communication as an integral part of inclusive crisis communication.

### Recommendation

Based on our results, we suggest the following guidelines for inclusive public policy, and the handling of disasters and health crises.

- Build relationships and communication channels with CSOs before a health crisis, by identifying key persons and organisations and building trust. Invite CSOs to be part of communication, to support the building of a knowledge bank and linguistically, culturally and contextually adapted communication channels and strategies, to be implemented in times of crisis. This should be supported by pre-crisis agreements (e.g., memoranda of understanding), joint training, and dedicated funding to sustain CSO capacity between crises.
- Include faith-based CSOs in crisis management, as they often hold a central position within their communities and, if excluded, become an obstacle when effective communication is needed. Include diversity in health crisis management to facilitate adaptation and trust of communication messages, and to ensure that exposed groups become an integral part of the overall crisis management effort, facilitating a shared sense of “we.” Strategies for inclusive intercultural crisis communication must be included in pandemic-preparedness plans and crisis-leadership structures.

## Data Availability

The raw data supporting the conclusions of this article will be made available by the authors, without undue reservation.
